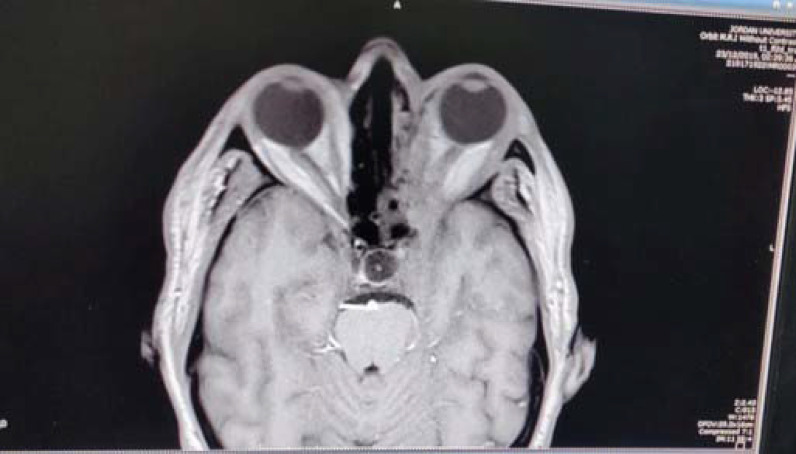# Mucormycosis with extensive cranial nerve involvement as the first presentation of diabetes mellitus: A case report

**DOI:** 10.5339/qmj.2021.61

**Published:** 2021-10-30

**Authors:** Mouna Al Saad, Ahmad Rimawi, Ahmad Saadeh, Amin Shehadeh

**Affiliations:** ^1^Department of Special Surgery, School of Medicine, University of Jordan, Amman, Jordan E-mail: eye_maas@yahoo.com; ^2^School of Medicine, University of Jordan, Amman, Jordan; ^3^Primary Health Care Corporation, Doha, Qatar

**Keywords:** Mucormycosis, diabetes, cranial nerve palsies, cavernous sinus thrombosis

## Abstract

Mucormycosis, a rare fungal infection, mainly affects individuals with diabetes mellitus and those who were immunocompromised and has a high mortality rate. Its most common presentation is similar to that of acute bacterial sinusitis with symptoms of nasal congestion, headache, and fever. The involvement of multiple cranial nerves in mucormycosis was rarely reported in the literature and indicates severe disease. Herein, we report the case of a 56-year-old man who was referred to the ophthalmology outpatient clinic for facial nerve palsy. He was treated with systemic steroids for 10 days with no improvement. On examination, he had a loss of vision and a frozen orbit due to involvement of cranial nerves II, III, IV, V, VI, and VII. An extensive workup revealed a hemoglobin A1C of 10%. However, he was never diagnosed with diabetes mellitus previously and denied any of the classical symptoms of diabetes mellitus. He underwent ethmoidectomy, maxillectomy, and drainage of an intraorbital abscess after appropriate imaging studies. Histopathology confirmed the diagnosis of mucormycosis, and the patient was started on systemic amphotericin B. This case emphasizes the importance of screening for diabetes mellitus. Early recognition of underlying diabetes mellitus in this patient may have prevented the development of mucormycosis along with its devastating complications.

## Introduction

Mucormycosis is a rare opportunistic angio-invasive infection caused by fungi of the order Mucorales. It mainly affects individuals with diabetes mellitus and patients who have underlying immunosuppression, with a mortality rate reaching up to 80%.^1^According to reports from the United States, mucormycosis has a very low incidence of 1.7 in every million individuals. Diabetes mellitus is the most common risk factor for the development of mucormycosis across Asia, while hematological malignancies are the major risk factors across Western countries.^2^ Although mucormycosis is a severe complication of poorly controlled diabetes mellitus, it is very rare for diabetes mellitus to be diagnosed after the development of mucormycosis.^3^ Mucormycosis can involve many organ systems, including the cutaneous, pulmonary, and gastrointestinal systems, and in severe forms, it can spread to other organs. However, rhinocerebral involvement is the most frequent presentation.^4^ Involvement of cranial nerve VII along with the widespread involvement of other cranial nerves was rarely reported in the literature.^5^ Herein, we report a very unusual case of mucormycosis with extensive rhino-orbit cerebral involvement presenting with multiple cranial nerve palsies as a first presentation of diabetes mellitus in a previously healthy patient.

## Case Description

A 56-year-old man was referred to our institution for a case of facial palsy. He was treated with systemic methylprednisolone for 10 days, without improvement. On examination, he had a visual acuity of 1.0 in the right eye and hand motion in the left. Motility examination revealed limitation of abduction of the right eye. The patient had a frozen orbit with a limitation of all gazes in the left eye. Fundus examination revealed a pale optic disk, afferent pupillary defect, and an ischemic retina, indicating central retinal artery occlusion. Examination findings were also consistent with oculomotor, trochlear, and abducens nerve paresis. Mucormycosis was a result of steroid therapy, as the patient was diagnosed 2 weeks previously with an isolated cranial nerve VII and was started with steroids in a peripheral ophthalmology clinic. At his first presentation in Jordan University Hospital, he had a frozen orbit with cranial nerve III, IV, and VI palsies.

The patient was admitted, and the results of baseline investigations were normal, except for a high white blood cell count and hemoglobin A1C (HbA1c) of 10%. Systemic steroids were stopped, and the patient was started on insulin to correct his blood sugar. He was never diagnosed with diabetes mellitus before and denied any symptoms of diabetes mellitus. He was initially diagnosed with diabetes mellitus. He tested negative for ketone bodies and was treated in the ward. Results of other routine investigations, such as kidney function tests and complete blood count, were normal, except for high blood sugar and a high white blood cell count.

The differential diagnosis of his current symptoms was cavernous sinus thrombosis, a space-occupying lesion in the cavernous sinus or orbital cellulitis.

**Figure 1.**
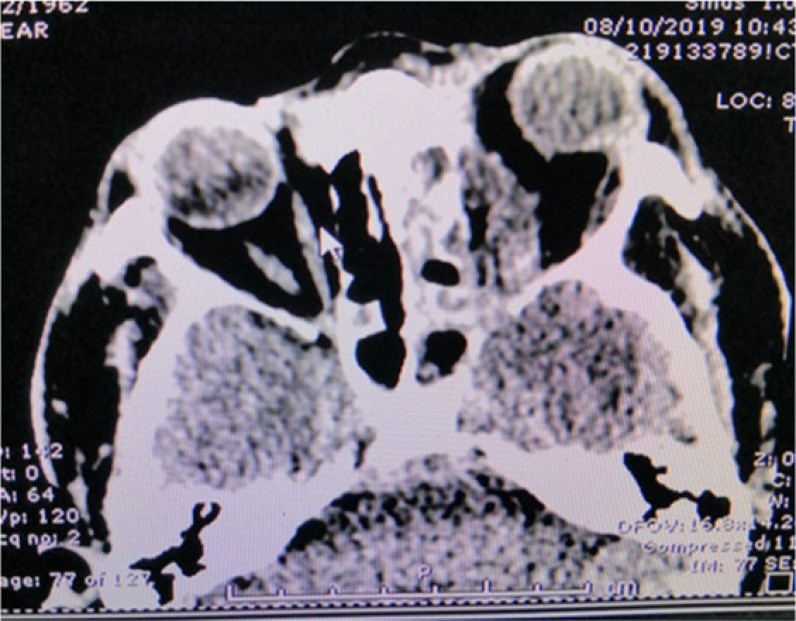


Computed tomography of the brain and orbit was performed and revealed extensive sinus disease with left complicated ethmoidal and maxillary sinusitis extending into the left orbit with abscess formation. There was also extensive orbital cellulitis and evidence of left cavernous sinus thrombosis secondary to infection (Figure 1).

It is possible that the patient had undiagnosed diabetes mellitus as indicated by a high HbA1c and was worsened by steroid therapy

The patient was started on systemic vancomycin and ceftriaxone, and a swab from the nasal cavity was sent for evaluation. Nasal swab revealed methicillin-resistant staphylococcus and positive fungal staining. The patient was started on systemic amphotericin B at a loading dose and continued with the therapeutic dose after 2 days of his presentation to our institution. Nasal endoscopy revealed diffuse white filaments and necrosis of the bone from which a biopsy was taken. Sinuses were drained, followed by ethmoidectomy and maxillectomy. A biopsy from the necrotic tissue was sent, which showed the presence of non-septate hyphae branching at right angles consistent with angio-invasive mucormycosis. However, workup to identify the exact causative organism of mucormycosis was not performed as it did not affect further management.

The patient was kept on daily irrigation of the sinuses along with administration of systemic amphotericin B. On follow-up magnetic resonance imaging, the patient had an intraorbital collection (Figure 2). Subsequently, drainage of the orbital abscess, tarsorrhaphy, and excision of the necrotic conjunctiva were performed. The patient was kept on systemic amphotericin B and showed clinical improvement. He remained hospitalized until a repeat biopsy of the paranasal sinuses was negative for the fungi. He was discharged with residual paresis of all cranial nerves involved along with left eye ptosis and only hand motion in the left eye. He was followed up serially for 1 year and 4 months. Currently, the patient is afebrile with complete resolution of symptoms in the right eye and partial recovery of cranial nerve palsies. However, in the left eye, residual ptosis has remained and only hand motion is visible due to the previous central retinal artery occlusion related to his initial presentation.

## Discussion

Mucormycosis is an opportunistic infection with a high mortality rate, reaching up to 80% without treatment.^6^ Even with early diagnosis and aggressive therapy, its prognosis remains poor.^7^ Diabetes mellitus is the most common risk factor for mucormycosis, particularly during ketoacidosis. Ketones induce the fungi to utilize and produce ketoreductase, which facilitates its growth through various mechanisms.^4^ Roden et al. reviewed the characteristics of 929 patients with mucormycosis, and of 929 patients, 36% had diabetes mellitus, 17% had an underlying malignancy, and 19% had no underlying conditions.^3^ In a review of literature of 851 patients, the median age was 51 ± 12 years, and 531 (63%) were men. Diabetes mellitus was the most common underlying condition (340/851, 40%; 71 (20%) had documented ketoacidosis), followed by hematological malignancy (275/851, 32%; 116 (42%) had acute myeloid leukemia) and solid organ transplantation (116/851, 14%; 67 (58%) had received a kidney transplant). Of the predisposing factors, the use of corticosteroids at the time of presentation was the most common (273/851, 33%), followed by neutropenia (169/851, 20%) and trauma (166/851, 20%). Rhino-orbito-cerebral mucormycosis was the most commonly observed manifestation (288/851, 34%), followed by cutaneous (187/851, 22%) and pulmonary mucormycosis (172/851, 20%.^8^ In the present case, the initiation of glucocorticoids as a treatment for facial nerve palsy may have facilitated the initial development of mucormycosis. Glucocorticoids are well known to increase blood glucose levels and can exacerbate hyperglycemia in a patient with diabetes mellitus.^9^ Our patient should have been tested for hyperglycemia before the initiation of glucocorticoid therapy (1) to rule out diabetes mellitus as the underlying cause of his facial palsy and (2) to measure the baseline blood sugar before the initiation of steroid therapy.^10^

Clinically, mucormycosis is characterized by rhinitis with granular and purulent discharge, nasal ulceration, black spots of infarcted mucosa, and paranasal sinusitis. However, other common presenting symptoms include epistaxis, ophthalmoplegia with blindness, proptosis and orbital cellulitis, hemiplegia or stroke, and decreased mental function.^11^ In advanced disease, symptoms include chemosis, ptosis, proptosis, ophthalmoplegia, blindness, and multiple cranial nerve palsies (function of cranial nerves II, III, V, VI, and VI may be lost or impaired).^4,5^ The classical clinical presentations are facial pain, an irregular black eschar on the palatal or nasal mucosa, and pus discharge from the eye and nose. In a retrospective chart review of 48 patients with 49 cases of acute fulminant-invasive fungal sinusitis over 19 years, mucormycosis and aspergillosis were found in 22 and 27 cases, respectively. Orbital (proptosis, periorbital edema, and ophthalmoplegia) and cranial nerve symptoms were seen at presentation more frequently in mucormycosis (6 [27%] and 9 [41%]) than in aspergillosis cases (3 [11%] and 7 [26%]; *p* = 0.079). Long-term orbital and cranial nerve sequelae were reported in 16 (72%) mucormycosis cases and 10 (37%) aspergillosis cases (*p* = 0.0210). These data suggest that the presence of orbital and neurological symptoms at presentation warrants more aggressive surgical intervention because of the likelihood of mucormycosis.^12^ In another review of mucormycosis cases, all six patients had uncontrolled diabetes mellitus with fungal-invasive disease and had multiple cranial nerve involvement, including cranial nerves II to VII.^2^ However, our patient had not been definitely diagnosed with diabetes mellitus before he had the fungal infection. That cohort chiefly complained of facial swelling, blood-stained nasal discharge, and crusting. All six patients were on prolonged antibiotic treatment with an incomplete response. Facial dysesthesia was present in all sides involved. Six patients had impaired vision, two had vision loss, and four only had a perception of light. These patients improved to finger counting during treatment. All patients had periorbital edema, ptosis, and 7^th^ cranial nerve palsy of the lower motor type, and two patients had irritability and disoriented state and succumbed to the disease within 48 h of treatment. By contrast, our patient was fully oriented and had an initial visual acuity of hand motion with limited improvement.^13^

A diagnosis of rhino-orbito-cerebral mucormycosis requires high clinical suspicion, and a definitive diagnosis requires a biopsy indicating the presence of the fungi. Furthermore, imaging is required to identify the degree of local involvement and spread across the rhino-orbital structures.^3^

Mucormycosis is treated with surgical debridement of necrotic tissues, use of intravenous antifungal drugs, and elimination of predisposing factors. In retrospective studies, the combination of surgery and antifungal drugs showed higher survival rates than medical treatment alone.^3^

Our case emphasizes the importance of screening for diabetes mellitus to prevent such a devastating complication. Our patient denied any of the classical symptoms of diabetes mellitus, and the only risk factor he had for diabetes mellitus was his age. Given the relatively long asymptomatic period of diabetes mellitus, the American Diabetes Association recommends screening for diabetes mellitus at age 45 years.^14^ Early detection and treatment of this patient's underlying diabetes mellitus may have prevented the development of mucormycosis, especially in a country like Jordan where the prevalence of diabetes mellitus among men aged >25 years is as high as 33%.^15^

The involvement of cranial nerve VII in mucormycosis is very rare, and its pathogenesis is not well understood.^13^ We are unsure whether the involvement of cranial nerve VII in this patient was the first manifestation of mucormycosis or it was due to an independent pathology in which its treatment with glucocorticoids facilitated the development of mucormycosis. Furthermore, the widespread involvement of cranial nerves III, IV, and VI is also uncommon and indicates severe disease possibly due to cavernous sinus thrombosis.^3^

## Conclusions

Mucormycosis is a rare and possibly fatal opportunistic infection. It should be suspected in any patient who presents with symptoms of sinusitis along with the widespread involvement of the orbit and cranial nerves. Workup should be initiated for an underlying systemic disease, such as diabetes mellitus, in any patient diagnosed with mucormycosis. In the present case, initiation of steroid therapy may have influenced the development of fungal infection and its progression, and this highlights the importance of screening. It is critical to exclude diabetes mellitus before starting steroid treatment and to screen a susceptible population perhaps at a younger age than recommended. Treatment with systemic antifungals, surgical debridement of necrotic tissues, and correction of underlying hyperglycemia should be initiated as soon as possible in diabetic patients with mucormycosis. Although this patient was 56 years old, screening for diabetes mellitus in younger populations earlier than 45 years is recommended, especially in the presence of opportunistic infections.

### Ethical statement

Ethical approval was obtained from the Jordan University Board. Informed consent was also obtained from the patient.

### Conflict of interest

All authors declare no conflict of interest

### Funding sources

This work was not funded by any funding bodies, and all authors have read and approved the contents of this manuscript.

## Figures and Tables

**Figure 2.**